# The chromosomal constitution of fish hybrid lineage revealed by 5S rDNA FISH

**DOI:** 10.1186/s12863-015-0295-8

**Published:** 2015-12-03

**Authors:** Chun Zhang, Lihai Ye, Yiyi Chen, Jun Xiao, Yanhong Wu, Min Tao, Yamei Xiao, Shaojun Liu

**Affiliations:** Key Laboratory of Protein Chemistry and Fish Developmental Biology of the Ministry of Education of China, College of Life Sciences, Hunan Normal University, Changsha, 410081 China

**Keywords:** Interspecific hybridization, Heterozygous chromosomes, FISH, 5S rDNA

## Abstract

**Background:**

The establishment of the bisexual fertile fish hybrid lineage including the allodiploid and allotetraploid hybrids, from interspecific hybridization of red crucian carp (*Carassius auratus* red var. 2n = 100, 2n = AA) (♀) × common carp (*Cyprinus carpio* L. 2n = 100, 2n = BB) (♂), provided a good platform to investigate genetic relationship between the parents and their hybrid progenies.

**Results:**

The chromosomal inheritance of diploid and allotetraploid hybrid progenies in successive generations, was studied by applying 5S rDNA fluorescence in situ hybridization. Signals of 5S rDNA distinguished the chromosomal constitution of common carp (B-genome) from red crucian carp (A-genome), in which two strong signals were observed on the first submetacentric chromosome, while no major signal was found in common carp. After fish hybridization, one strong signal of 5S rDNA was detected in the same locus on the chromosome of diploid hybrids. As expected, two strong signals were observed in 4nF_3_ tetraploid hybrids offspring and it is worth mentioning that two strong signals were detected in a separating bivalent of a primary spermatocyte in 4nF_3_. Furthermore, the mitosis of heterozygous chromosomes was shown normal and stable with blastular tissue histological studies.

**Conclusions:**

We revealed that 5S rDNA signal can be applied to discern A-genome from B-genome, and that 5S rDNA bearing chromosomes can be stably passed down in successive generations. Our work provided a significant method in fish breeding and this is important for studies in fish evolutionary biology.

## Background

In general, interspecific hybridization and polyploidy in plants were potent evolutionary mechanisms [1, 2]. Recently, new genetic evidence suggested that hybrid speciation was more common in plants and animals than we thought, and it has played a very constructive part in animal evolution [3, 4]. As fertile hybrids, the integrity and inheritance of heterozygous genome was a focused issue, which concerned to mitotic and meiotic stability of hybrid offspring and formation of fertile progenies. Now the inheritance rule of heterozygous genome had been widely studied in hybrid plants, while as there was a limit to the material, related research only focused on such a few hybrid vertebrates as fish and frog [5, 6].

Through selecting and breeding for more than 20 years, the bisexual fertile allotetraploid hybrid fish (abbreviated as AT) (4n = 200) has been acquired, which resulted from fertilization of unreduced eggs and sperm produced by hybrids of red crucian carp (*Carassius auratus* red var. 2n = 100) (♀) (abbreviated as RCC) × common carp (*Cyprinus carpio* L. 2n = 100) (♂) (abbreviated as CC). It provided a unique material for exploring the inheritance rule of heterozygous chromosomes in hybrid lineage [7–10]. The production procedure of AT was as follows: RCC females were mated with CC males to produce F_1_ fish (abbreviated as 2nF_1_) which were then mated with each other to produce F_2_ fish (abbreviated as 2nF_2_). The males and females of diploid F_2_ hybrids could generate unreduced diploid eggs and diploid sperm, respectively, which were fertilized to form the allotetraploid hybrids in F_3_ fish (abbreviated as 4nF_3_). Fertile tetraploid female and male F_3_ fish were found to generate diploid eggs and sperm, respectively. By self-breeding of F_3_, the tetraploid F_4_ fish were produced. Until now, the F_3_–F_24_ AT has been formed through successive breeding. Our previous study has indicated that the AT has inherited large amount of genetic material from their original parents with some variations [11–15]. However, the chromosomes constitution and inheritance pattern of hybrid lineage were still unknown. Identification of all genome chromosomes was crucial to understanding the genome constitution and inheritance rule of the hybrid genome. It has been widely studied in hybrid plant, using by fluorescence in situ hybridization (abbreviated as FISH), and genomic in situ hybridization (abbreviated as GISH) [16–18]. However, due to the lack of chromosome-specific molecular probes, the current reported studies were few in hybrid fish related to chromosomal localization.

The 5S rDNA in higher eukaryotes was organized in tandem repeat units that consisted of highly conserved 120 bp transcribing sequence and variable non-transcribed spacers (abbreviated as NTS) [19]. Variations in NTS, related to insertions-deletions, minirepeats and pseudo genes, were often species specific and have successfully been served as markers in evolutionary studies [20–22]. In addition, due to the numerous copies of repeated sequences, their chromosomal localization was easily detected by FISH. Analysis of 5S rDNA sequences and chromosomal localization of them were an effective method of genetic diversity monitoring, which were widely used to explore the phylogeny relationship among closer species and polyploidy origin [23–25]. Masaru and Hideo [26] also found that the 5S rDNA could be candidates for phylogenetic molecular markers for the crucian carp.

In the current study, based on our establishment of 2nF_1_, 2nF_2_, 4nF_3_ and 4nF_22_ hybrid lineage of RCC × CC, a comparative analysis of 5S rDNA fragments among all samples were carried by PCR and related sequences were screened out as probes to determine the chromosomal localization of the 5S rDNA for 2nF_1_, 2nF_2_, and 4nF_3_ fish by FISH, which elucidated the inheritance rules of heterozygous chromosomes in course of polyploid hybrid fish origin and propagation. Furthermore, cytological observation of early embryos in blastulastage was used to determine the mitotic stability in hybrid offspring.

## Methods

### Ethics statement

All experiments were approved by Animal Care Committee of Hunan Normal University and followed guidelines statement of the Administration of Affairs Concerning Animal Experimentation of China. All samples are raised in natural ponds and all dissections are performed under sodium pentobarbital anesthesia, and all efforts are made to minimize suffering.

### Fishes and genomic DNA samples

Specimens of RCC (2*n* = 100), CC (2*n* = 100), 2nF_1_ (2n = 100), 2nF_2_ (2n = 100), 4nF_3_ (4n = 200) and 4nF_22_ (4n = 200) were obtained from the Engineering Center of Polyploid Fish Breeding of National Education Ministry located at Hunan Normal University. Total genomic DNA was isolated from the peripheral blood cells according to the standard phenol-chloroform extraction procedures described by Sambrook et al. [27] with minor modifications.

### PCR amplification, cloning and sequencing

One pair of primers (5’-TATGCCCGATCTCGTCTGATC-3’ and 5’- CAGGTTGGTATGGCCGTAAGC-3’) [26] was synthesized to amplify the 5S rDNA repeats directly from genomic DNA by PCR. The genomic DNA of RCC, CC, 2nF_1_, 2nF_2_, 4nF_3_ and 4nF_22_ were used as a template for subsequent PCR (2 to 10 individuals for each of the 6 samples). The PCR cycling conditions were: 5 min at 94 °C, 30 cycles of denaturation at 94 °C for 30s, annealing at 56 °C for 30s, and extension 72 °C for 1 min, ending with 10 min of extension at 72 °C. The PCR products were analyzed in 1.2 % agarose gels stained by ethidium bromide, purified by Gel Ex- traction Kit (Sangon), cloned into the pMD18-T vector (Takara), and transferred into *E. coli* DH5α. Then the positive clones were sequenced by Sangon. The sequences were analyzed by ClustalW2.

### Chromosome preparation

The kidney cells in RCC, CC, 2nF_1_, 2nF_2_ and 4nF_3_ were used for the chromosome observation at the metaphase of mitosis and the testis cells in 4nF_3_ were used to observe the process of meiosis. The concanvalin A was injected into the abdominal cavity of the samples for one to three times at the dosage of 2-8 μg/g. The interval time was 12–24 h. Two to six hours prior to harvest, colchicines (2-4 μg/g) were used to arrest the chromosome at the metaphase. All the kidney tissue in the above samples was ground in 0.8 % NaCl. The hypotonic treatment was accomplished with 0.075 mol/L KCl for 40–60 min, followed by fixation in 3:1 methanol-acetic acid (three changes). The cells were spread on clean slides.

### Probe preparation of the 5S rDNA sequence and fluorescence in situ hybridization

FISH is used to assess chromosomal location of 5S rDNA of RCC, CC, 2nF_1_, 2nF_2_, and 4nF_3_ fish. The hybridization was performed according to the method described by Masaru and Hideo with minor modifications [26]. In brief, the purified PCR product of related 5S rDNA fragments of RCC labeled with Dig-11-dUTP by PCR DIG Probe Synthesis Kit (Roche, Germany) were used as probes. After pretreatment with 2 × SSC for 30 min, 70 %, and 100 % ethanol for 5 min each, the slides with chromosome metaphase spreads of all samples were denatured in 70 % deionized formamide /2 × SSC for 2 min at 75 °C, dehydrate in a 70 % (−20 °C, to avoid DNA renaturation) and 100 % ethanol series for 5 min each, and then air-dry. The probe was prepared by adding labeled DNA with 20 × SSC, deionized formamide and 50 % dextran sulphate and denaturing in boiling water for 5 min. Hybridizations were allowed to proceed under a sealed cover slip in a moist chamber at 37 °C overnight. The next day the slides were washed; twice for 15 min in 2 × SSC with 50 % formamide and then in 2 × SSC and 1 × SSC for 5 min each. After a series of post-hybridization washing were performed, the spectrum signals were achieved with 10ul of 1ug ml^−1^ FITC-conjugated anti-digoxigenin antibody from sheep (Roche, Germany). Then the slides were washed three times for 5 min each in TNT (formulas of eluate: 0.1 M Tris–HCl, 0.15 M NaCL,0.05 % Tween 20). After slides were placed in 10ul anti-fade solution containing 0.5 μg/ml of 4, 6-diamidino-2-phenylindole (abbreviated as DAPI), they were viewed using a Leica inverted DMIRE2 microscope image system (Leica, Germany). Images were captured with CW4000 FISH software (Leica, Germany). Good-quality metaphase spreads were photographed and used for analysis of karyotypes.

### Cytological observations of early embryos

After 6 h after fertilization, the F_3_ embryos in blastulastage were fixed in Smith solution for 4 to12 h, and then are washed by alcohol for 2 to 3 times. The paraffin-embedded sections were cut at 6 μm and stained with Harris hematoxylin and eosin. The structure of zygote was observed under a light microscope and photographed with Pixera Pro 600 ES.

## Results

### Analysis of 5S rDNA fragments

**Fig. 1:**
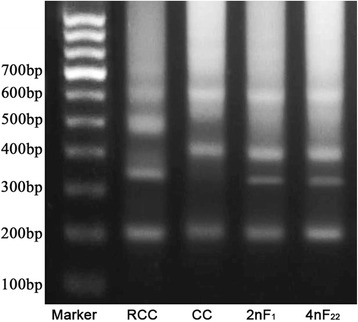
DNA bands amplify from RCC, CC and their hybrid offspring. Marker: DNA ladder markers (100 bp increments); RCC: two DNA bands (200, 340 bp) and some repeated ladder-like bands; CC: two DNA bands (200, 400 bp) and some repeated ladder-like bands; 2nF_1_ and 4nF_22_: three DNA bands (200, 340 and 400 bp) and some repeated ladder-like bands

The electrophoretic band pattern of DNA fragments amplified with the primers of 5 s rDNA was distinctive between RCC and CC. RCC exhibited two major DNA bands (approximately 200 bp and 340 bp) and some repeated ladder-like bands, CC exhibited other two major DNA bands (approximately 200 bp and 400 bp) and some repeated ladder-like bands as well. As their hybrid offspring, 2nF_1_ and 4n F_22_ exhibited three major DNA bands (approximately 200 bp, 340 bp and 400 bp) and some repeated ladder-like bands (Fig. 1). After ligation of sized DNA fragments and transformation, a total number of 100 clones were sequenced to examine the different patterns of 5SrDNA, including 30 clones from 5 RCC individuals, 20 clones from 5 CC individuals, 20 clones from 5 2 nF_1_ individuals and 30 clones from 5 4 nF_22_ individuals. consensus sequences of the 5 s rDNA repeat units were obtained. All of the typical sequences were deposited at GenBank under the accession numbers (KM359661- KM359679) (Table 1).

**Table 1:** Size (bp), type, and GenBank accession number for each of the 5SrDNA repeat units (coding sequence plus NTS) found in RCC, CC, 2nF_1_, and 4nF_22_

Species	Length(bp)			Accession no.
	5 s repeat	Coding sequence	NTS	
RCC	201	119	82 = 0*138 + 82^**RCC-a**^	KM359661
	202	120	82 = 0*138 + 82^**RCC-b**^	KM359662
	340	121	219 = 1*137 + 82	KM359663
	339	121	218 = 1*136 + 82	KM359664
	477	121	356 = 2*137 + 82	KM359665
	613	120	493 = 3*137 + 82	KM359666
CC	202	120	82 = 0*138 + 82^**CC-a**^	KM359667
	204	121	83 = 0*138 + 83^**CC-b**^	KM359668
2nF_1_	203	121	82 = 0*138 + 82	KM359671
	201	120	82 = 0*138 + 82	KM359672
	341	120	219 = 1*137 + 82	KM359673
	339	120	219 = 1*137 + 82	KM359674
4nF_22_	201	119	82 = 0*138 + 82	KM359675
	202	120	82 = 0*138 + 82	KM359676
	338	119	219 = 1*137 + 82	KM359677
	477	120	357 = 2*138 + 82	KM359678
	639	122	504 = 3*137 + 93	KM359679

^RCC-a, RCC-b, CC-a, CC-b^: indicate the different approximately 82 bp-units. The alignment data of them was showed in Table 2

Nucleotide sequencing and BlastN sequences corresponded to 5 s rDNA repeat units. All units consist of a 120 bp coding region and a variable NTS. In RCC, four kinds of fragments of 5 s rDNA (202 bp, 340 bp, 477 bp and 613 bp) were characterized by different lengths of NTS. In CC, there was only one kind of fragment of 5 s rDNA (202 bp) with 120 bp coding region and one 82 bp NTS unit. The 400 bp fragment from amplified DNA bands was just two repeats of 202 bp unit. In 2nF_1_ and 4nF_22_, like their original parents, two kinds of fragments of 5 s rDNA (approximately 200 bp and 340 bp) both contained similar coding region and NTS (Table 1).

**Fig. 2:**
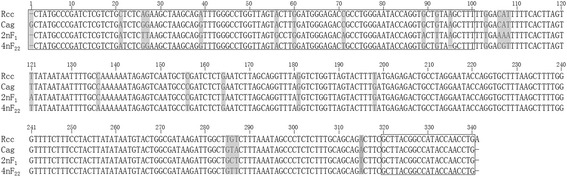
Sequence alignments of 340 bp-fragments of RCC, Cag, 2nF_1_ and 4nF_22_. The 120 bp coding regions were included in the boxes. The sequence data of RCC, Cag, 2nF_1_ and 4nF_22_ were available from GenBank under accession number KM359663, DQ659260, KM359673, KM359677

The NTS sequence of 5S rDNA also showed an extensive variation between RCC and CC. In RCC, different-sized 5S rDNA units contained one 120 bp coding region and one 82 bp repeat unit, which were spaced at regular interval with zero, one, two, or three 138 bp repeat units (Interposed Region: IPR), respectively showing 202 bp, 340 bp, 477 bp and 613 bp 5S rDNA repeat units. While in CC, only around 200 bp-sized 5S rDNA units consisting of one 120 bp coding region and one monomeric 82 bp repeat unit were identified. The similarity of 82 bp-unit among different clones of RCC was 95.1 % and the similarity of 82 bp-unit among different clones of CC was 91.5 %, while the 82 bp-unit of RCC was different from 82 bp-unit of CC with lower similarity of 52.4 %-68.3 % (Table 2). Sequence alignments showed high similarity of 340 bp units between RCC, 2nF_1_ and 4nF_22,_ even in gibel carp, *Carassius auratus*(abbreviated as Cag) [25] (Fig. 2 and Table 3), so the 340 bp-fragments were screened out as a probe to assess chromosomal location of 5S rDNA of RCC, CC, 2nF_1_, 2nF_2_, and 4nF_3_ fish.

**Table 2:** Nucleotide similarities of 82 bp-units of 5S rDNA sequence between RCC and CC

Sequence name	RCC-a	RCC-b	CC-a	CC-b
RCC-a	-	95.1 %	68.3 %	53.7 %
RCC-b	-	-	68.3 %	52.4 %
CC-a	-	-	-	91.5 %
CC-b	-	-	-	-

**Table 3:** Nucleotide similarities of 340 bp- fragments among RCC, Cag, 2nF_1_ and 4nF_22_

sequence name	Cag-340	2nF_1_-340	4nF_22_-340
RCC-340	97.1 %	94.4 %	97.3 %
Cag-340	-	95.0 %	97.9 %
2nF_1_-340	-	-	95.6 %

### 5S rDNA localization

The diploid RCC, CC, 2nF_1_, and 2nF_2_ samples showed 100 chromosomes which were classified into 22 metacentric (abbreviated as M), 34 submetacentric (abbreviated as SM), 22 subtelelocentric (abbreviated as ST) and 22 telocentric (abbreviated as T) chromosomes. The tetraploid 4nF_3_ samples revealed 200 chromosomes, which were classified into 44 metacentric (M), 68 submetacentric (SM), 44 subtelelocentric (ST) and 44 telocentric(T) chromosomes [7, 28]. No difference was observed between the females and the males.

**Fig. 3:**
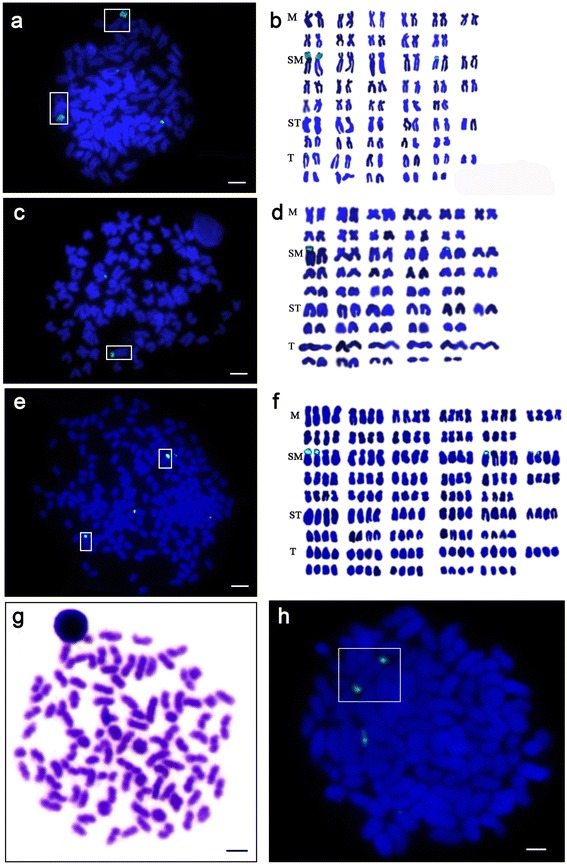
Mitotic metaphase spreads and karyotypes of RCC, 2 nF_2_ and 4 nF_3_ fish with FISH signals of 5S rDNA. (**a**) Two strong and a few weak signals were detected in RCC fish; the boxes indicated two major signals of 5S rDNA (green). (**b**) The karyotype of RCC fish revealed two strong signals located on the first pair of larger-sized SM chromosomes. (**c**) One strong signal and a few weak signals in 2nF_2_ fish (green),the box indicated the one major signal of 5S rDNA (green). (**d**) The karyotype of 2nF_2_ fish show one strong signal on the first group of larger-sized SM chromosomes. (**e**) The FISH analysis of 4nF_3_ fish exhibited the double signal feature of two strong and a few weak signals (green), the boxes indicated the two major signals (green). (**f **) The karyotype of 4nF_3_ fish showed two strong signals on the first group of larger-sized SM chromosomes. All metaphase chromosomes were counterstained with DAPI and appear blue. (**g**) The chromosome pairing in Meiosis I of spermatocytes in 4nF_3_ showed 100 bivalents under light microscope; (**h**) Two signals of 5S rDNA located on a separating bivalent. M: metacentric; SM: submetacentric; ST subtelelocentric; T: telocentric. (**a**-**f**) Bar = 3 μm; (**g**,**h**) Bar = 5 μm

Two signals of 340 bp-fragment were clearly detected in the first SM chromosomes pair of RCC, with a few weak signals being detected in other chromosomes (Fig. 3a, b), while the CC fish has no major signal. As to their hybrid lineage offspring, one strong signal was found in the first SM chromosome of 2nF_1_ and 2nF_2_ fish (Fig. 3c, d) and two strong signals were detected in the same chromosomes of AT (Fig. 3e, f), also with a few weak signals being detected in other chromosomes.

Chromosomal localization of 340 bp-fragment in metaphase of meiosis spreads in primary spermatocytes of 4nF_3_ indicated that two strong signals being located on a separating bivalent (Fig. 3g, h).

### Cytological observation of early embryos in blastulastage

**Fig. 4:**
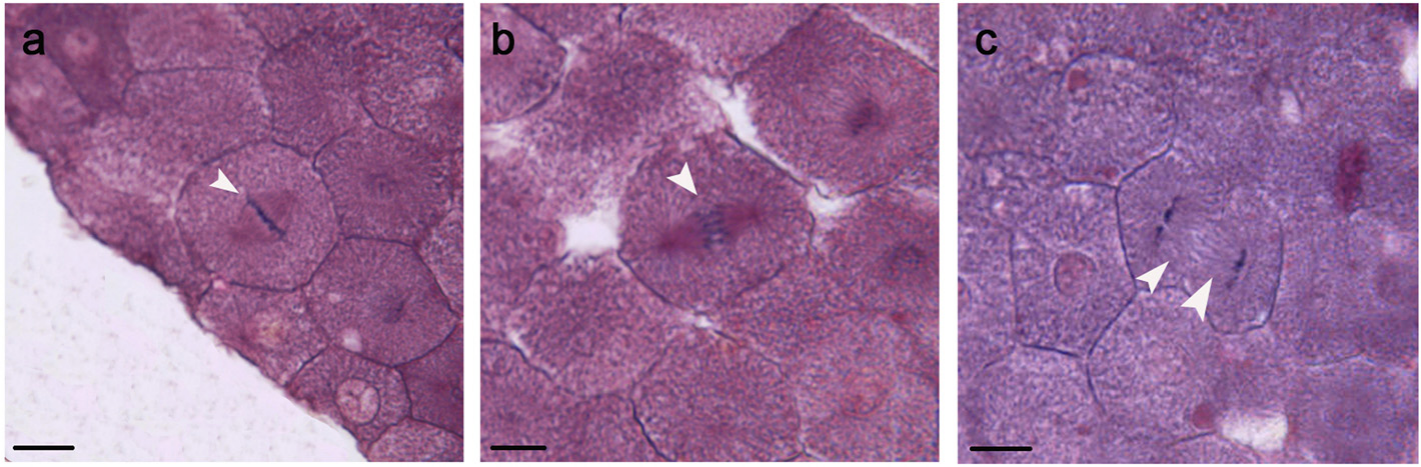
Cytological observations of early embryos in blastula stage. (**a**) Early embryo cells in metaphase of mitosis; (**b**) Early embryo cells in anaphase of mitosis; (**c**) Early embryo cells in telophase of mitosis; the arrowheads showed the normal distribution of chromosomes and structure of spindle body. Bar = 10 μm

Cytological observation of mitosis in F_3_ early embryo cells showed that F_3_ performed normal and stable mitosis, with normal distribution of chromosomes and structure of spindle body during metaphase (Fig. 4a), anaphase (Fig. 4b), and telophase (Fig. 4c) of mitosis, not being affected by the genetic attributes of heterozygous genome.

## Discussion

Different 5 s rDNA classes were reported in several mammals and fish species, and generally two different type of 5 s rDNA units have been characterized by distinct NTS types and base substitution [29, 30]. In our work, the construction of different-sized 5S rDNA units were further elucidated as containing one 120 bp coding region and one 82 bp repeat unit, which were spaced at regular interval with zero, one, two, or three 138 bp-IPR repeat units (Table 1), this result revealed the assembly rule of NTS in RCC, which enriched the current data of molecular organization analysis of 5 s rDNA.

Modern cytogenetic analyses, such as FISH and GISH have been widely applied for studies in many processes of chromosome evolution, including structural rearrangements, as well as extensive studies on phylogenetic and genomic relationships [31]. In this work, chromosome inheritance of hybrid lineage progenies of RCC × CC were investigated by detecting the signal of 340 bp fragments of 5 s rDNA on chromosomes in successive generations, i.e. RCC, CC, 2nF_1_, 2nF_2_, and 4nF_3_ fish. The 340 bp fragments was highly conserved between RCC and their hybrid lineage progenies, but not in CC genome, which made the 340 bp fragments in RCC a suitable indecator to monitor chromosomal inheritance in RCC, CC and their hybrid progeny, as it was recently showed in other hybrid fish [32, 33]. Besides that, our results revealed that the chromosomal localization of the 340 bp fragments of RCC could serve as a suitable genetic marker to distinguish the chromosomal constitution of CC from RCC, as two strong detected signals on the first pair of larger-sized SM chromosomes were observed, but not in CC chromosomes. The other weak FISH signals were also 5S rDNA locus with less copy of repeat units.

**Fig. 5:**
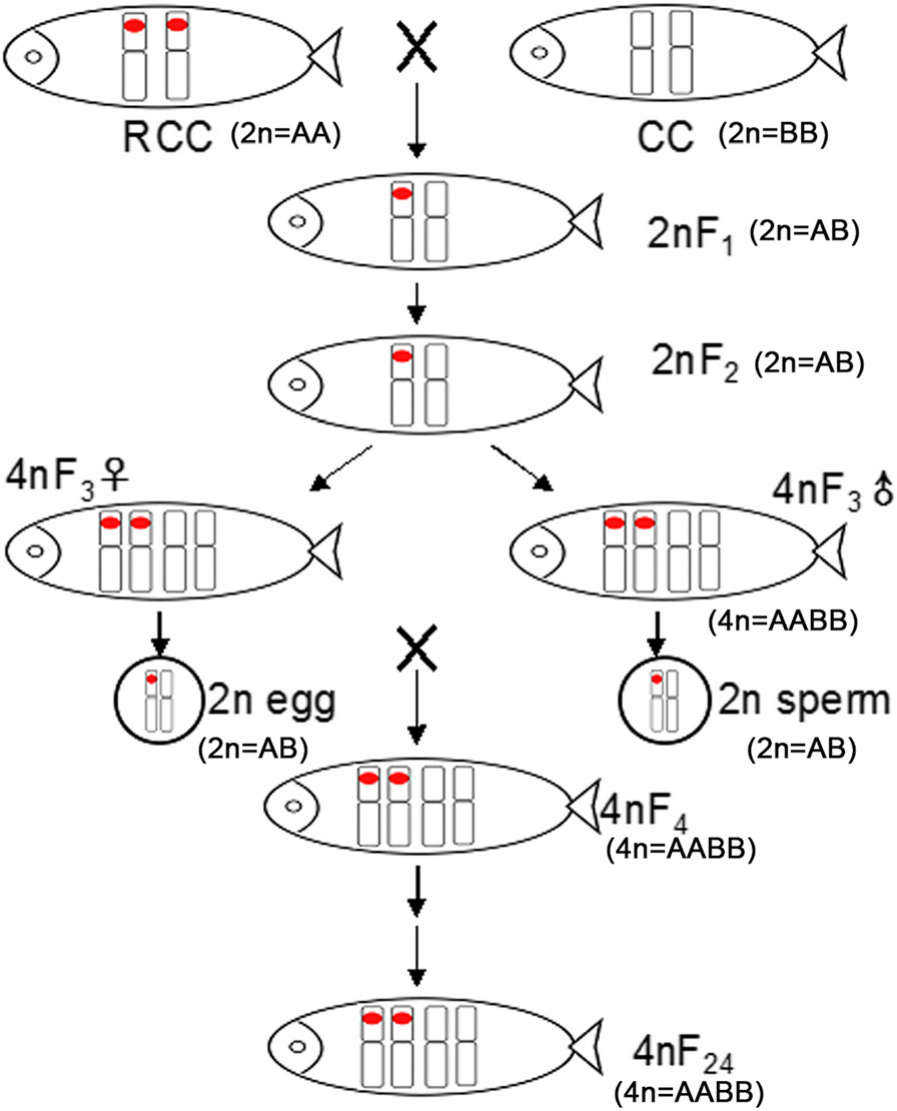
Schematic presentation of chromosome transmission in hybrid fish from RCC, CC to 2nF_1_, and to 2nF_2_, as well as to 4nF_3_ and 4nF_24_

Since there have been very few studies on chromosomal inherence behavior in fish hybridization, we looked into the 5S rDNA bearing chromosome in successive hybrid generation. The strong signal with same locus as observed in RCC were also found in 2nF_1_ and 2nF_2_ fish and the number of signal doubled in 4nF_3_ fish. Furthermore, two strong signals were also detected in a separating bivalent of a primary spermatocyte in 4nF_3_, which promised that two 5S rDNA sites would equally distributed to each gamete and made every gamete acquire one 5S rDNA bearing chromosome. Those results may suggest that 2nF_1_ and 2nF_2_ fish contained one 5S rDNA bearing chromosome from A-genome and one 5S rDNA bearing chromosome from B-genome, and 4nF_3_ fish possessed two 5S rDNA bearing chromosomes from A-genome and two 5S rDNA bearing chromosomes from B-genome. Additional studies was made in order to investigate the mitosis, results demonstrated that in hybridization the mitosis process maintained normal and stable, which demonstrated the compatibility of genome between RCC and CC, and promised the genetic stability of hybrid progeny. Based on the fish analysis and cytological observation, we speculate that chromosomes of RCC and CC may equally pass down to hybrid generation (Fig. 5).

The chromosomal rearrangements having occurred less frequently was a promising result supporting the development of stable allotetraploid hybrid lines, which were also proved in allohexaploid *Brassica* line [34] and the lineage of *X.laevis* [35]. However, the allotetraploid *Festuca pratensis* × *Lolium perenne* hybrid of three generations shared various rDNA loci profiles with chromosomal rearrangements [31], indicating a tendency of *F. pratensis* genome-like chromosomes to be less stable in hybrid of three generations. In our work, the strong FISH signals of 5S rDNA in RCC passed down stably in successive hybrid generation, which implied that chromosomal rearrangements have occurred less frequently in area of highly repeated sequence of 5S rDNA. Thus it can be seen that genomic contribution was different in different type of hybridized combinations, which will affect the genetic stability of hybrid progenies. Further studies were necessary to prove or reject this hypothesis as well as for deeper understanding of the mechanisms responsible for the fate of heterozygous chromosomes in hybrid lineage progenies.

## Conclusions

Few studies focused on chromosomal inheritance in successive generations of hybrid fish. We executed the 5S rDNA FISH analysis in successive generations of diploid and allotetraploid hybrid progenies and revealed 5S rDNA signal can be applied to discern A-genome from B-genome, and that 5S rDNA bearing chromosomes respectively coming from A-genome and B-genome can be stably passed down in successive hybrid generations.

## Abbreviations

AT: Allotetraploid hybrid fish
RCC: Red crucian carp
CC: Common carp
2nF_1_: The first generation of diploid hybrid fish of RCC × CC
2nF_2_: The second generation of diploid hybrid fish of RCC × CC
4nF_3_: The third generation of tetraploid hybrid fish of RCC × CC
Cag: Gibel carp, *Carassius auratus*
FISH: Fluorescence in situ h ybridization
GISH: Genomic in situ hybridization
NTS: Non-transcribed spacers
IPR: Interposed region
DAPI: 4, 6-diamidino-2-phenylindole
M: Metacentric
SM: Submetacentric
ST: Subtelelocentric
T: Telocentric
